# Editorial: Mechanisms behind stress tolerance induced by mycorrhizal symbioses

**DOI:** 10.3389/fpls.2025.1751899

**Published:** 2025-12-11

**Authors:** Qiang-Sheng Wu, Qing Yao, Xiancan Zhu, Abeer Hashem, Bhoopander Giri

**Affiliations:** 1 Hubei Key Laboratory of Spices & Horticultural Plant Germplasm Innovation & Utilization, College of Horticulture and Gardening, Yangtze University, Jingzhou, China; 2 Key Laboratory of Biology and Genetic Improvement of Horticultural Crops (South China), Ministry of Agriculture and Rural Affairs, Guangdong Province Key Laboratory of Microbial Signals and Disease Control, College of Horticulture, South China Agricultural University, Guangzhou, China; 3 Anhui Provincial Key Laboratory of the Conservation and Exploitation of Biological Resources, College of Life Sciences, Anhui Normal University, Wuhu, China; 4 Botany and Microbiology Department, College of Science, King Saud University, Riyadh, Saudi Arabia; 5 Department of Botany, University of Delhi, Delhi, India

**Keywords:** abiotic stress, arbuscule, low-temperature stress, mycorrhiza, heavy metal stress, drought stress

Plants are consistently challenged by a wide range of biotic and abiotic stresses, which severely limit their growth and productivity (Ray et al., 2025). These stresses trigger a complex cascade of physiological and biochemical responses within the plants, leading to detrimental effects such as overproduction of reactive oxygen species, disruption of plasma membrane integrity, and RNA degradation (Siddique et al., 2024; Su et al., 2025). Ultimately, such disturbances compromise cellular homeostasis, resulting in reduced crop yield and, in severe cases, cell death.

Through mutualistic interactions with beneficial rhizosphere microorganisms such as arbuscular mycorrhizal (AM) fungi, plants receive water and mineral nutrients via the fungal extraradical hyphae network, while supplying carbohydrates (primarily in the form of fatty acids) to support AM hyphal growth (Luginbuehl et al., 2017; Gebisa, 2024). Arbuscular mycorrhizas are pivotal for enhancing plant growth and resilience, particularly under stress conditions (Zou et al., 2021). The aim of this Research Topic was to explore the mechanisms of mycorrhiza-induced stress tolerance and to identify strategies for advancing both understanding and practical applications of this symbiosis in improving plant stress resistance. A total of eight articles have been accepted for publication, six of which focus on AM fungi and two on ectomycorrhizal fungi, including one review.

Mycorrhizal plants possess multiple pathways to respond to and alleviate abiotic stresses (Cheng et al., 2021; Shi et al., 2023; Samanta et al., 2025). Beyond improving nutrient acquisition, AM symbiosis activates complex physiological, biochemical, and molecular mechanisms that collectively improve plant resilience under adverse environmental conditions. The study byYao et al. delves into the specific mechanisms by which *Funneliformis mosseae* improves the low-temperature tolerance of white clover. The study revealed that AM fungal inoculation significantly increased the content of putrescine, spermidine, and spermine in white clover under low-temperature stress, offering valuable insights into cold acclimation in forage crops. This fine-tuned regulation of polyamine metabolism by AM fungi appears to be a key mechanism underlying enhancing cold tolerance.

Although AM fungi generally enhance host plants’ stress tolerance, these stresses themselves can paradoxically impair the mycorrhizal functionality. Zhang et al. demonstrated that drought stress significantly impaired AM colonization and arbuscule abundance in trifoliate orange roots. This impairment directly disrupted lipid allocation, essential for fungal development and membrane integrity, thereby hindering plant growth via impaired lipid metabolism. Their study underscores the importance of lipid dynamics as a central regulatory node in the metabolic interaction between host plants and AM fungi. Understanding these mechanisms offers promising avenues for improving citrus resilience via targeted metabolic engineering or microbial interventions. In contrast, Zhang et al. provides compelling evidence that AM fungi, specifically *Rhizophagus irregularis*, confer a concentration-independent enhancement of vanadium stress tolerance in green foxtail. This finding deviates from conventional dose-response models, as the symbiosis consistently improved host performance across varying contamination levels by protecting leaf ultrastructure, increasing chlorophyll, alleviating oxidative damage, and diluting vanadium through enhanced biomass.

In addition to physiological responses, AM fungi elicit extensive molecular changes that help elucidate underlying mechanisms involved (Boyno et al., 2025). The study by Pan et al. demonstrated that the AM fungus *Claroideglomus etunicatum* significantly enhances arsenic resistance and accumulation in the hyperaccumulator *Pteris vittata*, primarily through the modulation of arsenic reduction (reduced compartmentalization of arsenic in roots and enhanced translocation to leaves) and transport mechanisms within plant roots through comparative transcriptomic analysis. Similarly, the study by Wang et al. also supported the use of specific ectomycorrhizal fungal strains as bio-inoculants in phytoremediation programs targeting cadmium-polluted landscapes. This research elucidated species-specific detoxification strategies in *Salix psammophila*: *Cenococcum geophilum* primarily bolstered general stress resilience through constitutive defense activation, while *Suillus luteus* induced a more dynamic, inducible response involving pathogenesis-related proteins and secondary metabolite synthesis. This functional specialization highlights the diversity of adaptive mechanisms among ectomycorrhizal fungi under cadmium stress.

Traditionally, spores have been the preferred propagules in AM fungal research and application due to their structural resilience and ease of quantification, although mycorrhizal extraradical mycelium and mycorrhizal root fragments also serve as inoculum sources (Varela-Cervero et al., 2016). However, the study by Liu et al. reported that cold storage not only preserves but also actively promotes the regenerative potential (germination and colonization capacity) of hyphal networks, suggesting that low temperatures may modulate physiological dormancy mechanisms. Hence, common cold storage protocols can be optimized to improve, rather than simply preserve, AM fungal functionality. The study by Zhang et al. addresses how long-term orchard management influences belowground ecology, particularly plant–AM fungal symbiosis. They demonstrates that long-term (20 years) loquat cultivation reshapes AM fungal community composition, reducing overall diversity and favoring stress-tolerant but functionally less efficient taxa. This compositional shift correlated with reduced root colonization rates and hyphal development, indicating a decline in symbiotic efficiency under sustained agricultural pressure.

As highlighted by Wang et al., ectomycorrhizal fungi also contribute to plant stress responses. In this regard, an integrated biological, genetic, and ecological examination of *Cenococcum geophilum*, a fungus renowned for its exceptional stress resilience, could provide a holistic perspective on its adaptive mechanisms (Shi et al., 2022). The study by Wang et al. emphasized the role of *C. geophilum* as a pioneer colonizer during primary succession and its dominance in extreme environments as arid, nutrient-poor, and contaminated soils. As a species complex composed of multiple cryptic lineages, *C. geophilum* exhibits substantial genetic variability, likely facilitating its adaptability and broad distribution across biomes from polar to tropical. The environmental prevalence of this fungus is particularly significant in an era of increasing climate change and drought frequency.

In summary, the studies included in this Research Topic analyze the dual roles and mechanisms of plant–mycorrhizal interactions in stress responses. The core insight is that AM fungi can significantly enhance plant tolerance to abiotic stresses by modulating key physiological and molecular pathways. However, the effectiveness of these benefits may be limited by the intensity of the stress itself and long-term agricultural practices. Together, these contributions offer valuable insights for deepening the understanding of the stress-resistance mechanisms conferred by mycorrhizas and advancing their practical applications.
